# 3D Printing in Pharmaceutical and Medical Applications – Recent Achievements and Challenges

**DOI:** 10.1007/s11095-018-2454-x

**Published:** 2018-07-11

**Authors:** Witold Jamróz, Joanna Szafraniec, Mateusz Kurek, Renata Jachowicz

**Affiliations:** Department of Pharmaceutical Technology and Biopharmaceutics, Faculty of Pharmacy, Jagiellonian University Medical College, Medyczna 9, 30-688 Krakow, Poland

**Keywords:** 3D printing, additive manufacturing, bioprinting, drug delivery, medical devices

## Abstract

Growing demand for customized pharmaceutics and medical devices makes the impact of additive manufacturing increased rapidly in recent years. The 3D printing has become one of the most revolutionary and powerful tool serving as a technology of precise manufacturing of individually developed dosage forms, tissue engineering and disease modeling. The current achievements include multifunctional drug delivery systems with accelerated release characteristic, adjustable and personalized dosage forms, implants and phantoms corresponding to specific patient anatomy as well as cell-based materials for regenerative medicine. This review summarizes the newest achievements and challenges of additive manufacturing in the field of pharmaceutical and biomedical research that have been published since 2015. Currently developed techniques of 3D printing are briefly described while comprehensive analysis of extrusion-based methods as the most intensively investigated is provided. The issue of printlets attributes, i.e. shape and size is described with regard to personalized dosage forms and medical devices manufacturing. The undeniable benefits of 3D printing are highlighted, however a critical view resulting from the limitations and challenges of the additive manufacturing is also included. The regulatory issue is pointed as well.

## Introduction

There is a constant motivation towards new concepts in drug design, better understanding of material properties, manufacturing technology and processes that assures high quality of dosage forms. The diversity of physicochemical and biopharmaceutical characteristics of active pharmaceutical ingredients (APIs) have to be considered and studied through each stage of product development. Auxiliary substances need to be examined as well in order to manufacture of the desired dosage form.

Within last decade the patient-centric drug product development has been under considerable attention. It was focused on novel dosage forms and technological processes. Growing demand for customized devices combined with an expansion of technological innovation drives the major progress in personalized medicine expressed e.g. by the production of small series of individually-selected doses and tailor-made prostheses meet the anatomical needs of patients. Within many discoveries introduced into pharmaceutical and biomedical market, three-dimensional printing (3DP) is believed to be the most revolutionary and powerful. This technique is recognized as a versatile tool of precise manufacturing of various devices. It serves as a technology for developing novel dosage forms, tissues and organs engineering as well as disease modeling.

Nowadays, three-dimensional printing is one of the fastest developing branch of technology, art and science, and still broadens the applications. The term three-dimensional printing was defined by International Standard Organization (ISO) as: “fabrication of objects through the deposition of a material using a print head, nozzle, or another printer technology”. In contrast to commonly used subtractive and formative manufacturing methodologies, this technique is one of the methods of additive manufacturing (AM) in which the parts are prepared from 3D model data in the process of joining materials layer by layer. The practical approach of AM is called rapid prototyping (RP) (1) and its advantages include the reduction of prototyping time and costs, easy modifications of a product at a designed level, the possibility of manufacturing of small objects, individualized product series or structures impossible to be formed with subtractive techniques (2).

The application of 3D printing in the science and engineering has grown since 2012. The number of scientific papers recorded in Web of Science Core Collection containing a term “3D printing” or “3D printed” in the title increased from 59 in 2012 to 1573 in 2017. Moreover, the number of citations of these papers in the same period grown from 209 to 12,411. Narrowing the searching results to the category of pharmacy/pharmacology gives no result in 2012, however 77 records were found up to 2017, which also shows a great interest in the 3DP methods in pharmaceutical sciences.

This review is aimed at the newest development and achievements in the field of pharmaceutical and biomedical research from the literature items that have been published within the last 3 years. The novel approaches in the formulation of solid dosage forms for individualized therapy are particularly focused, however transdermal drug delivery as well as biomedical applications of additive manufacturing technique including implants, surgical models, bioprinted materials and biorobotics are also mentioned. The parallel development of additive manufacturing used in pharmaceutical technology and bioprinting is summarized and compared with a special effort made to point out the evolution of bioprinting. Due to the fact that the pharmaceutical applications of additive manufacturing is on the early stage of development and implementation not many regulatory is available however the important issues that have been introduced by the FDA in 2017 are mentioned.

## A Bit of History

The idea of 3DP has evolved from early 70′ of the twentieth century when Pierre A. L. Ciraud described the method of application of powdered material and subsequent solidification of each layer through the action of high energy beam. In this case meltable materials such as plastics or metals can be theoretically used for object preparation. In early 80′ in a patent entitles: “A molding process for forming a three-dimensional article in layers”, Ross Housholder described an idea of sand binding by different materials and Carl Deckard developed a method of solidification of powdered bed by laser beam called selective laser sintering (SLS).

**Fig. 1:**
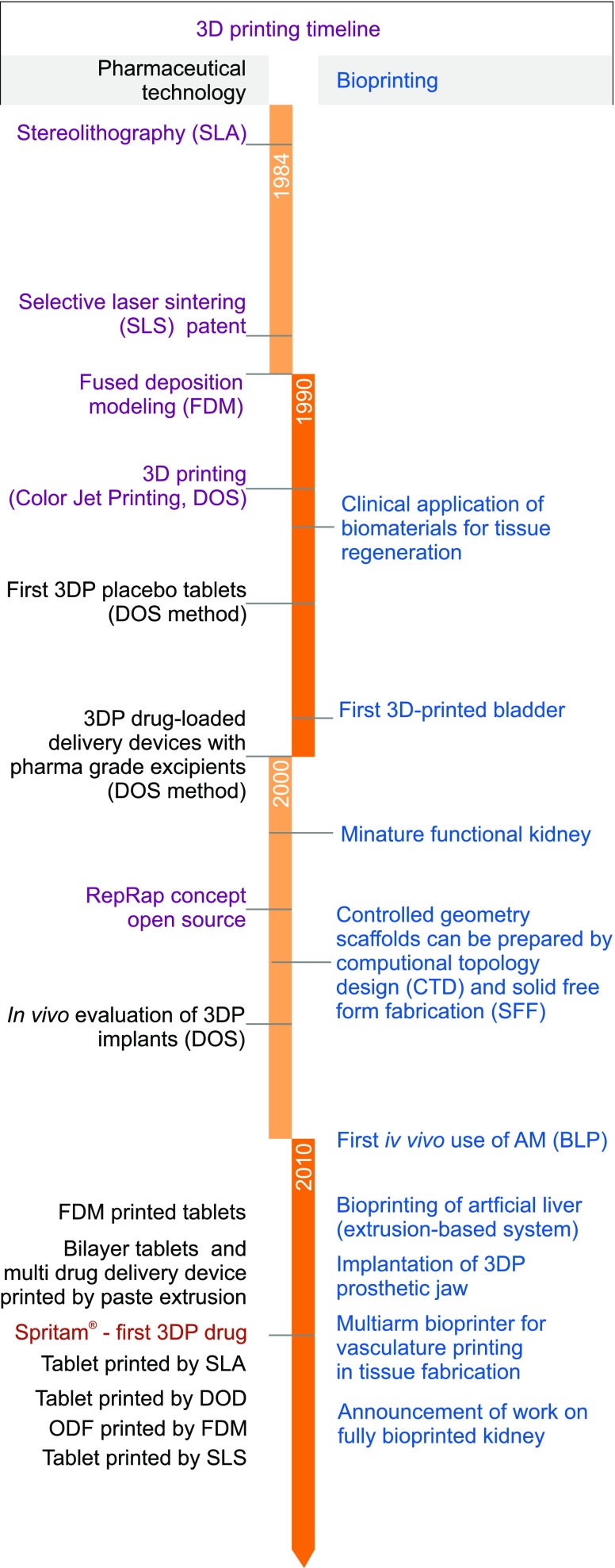
The most important achievements in 3D printing in pharmaceutical and biomedical applications.

The first commercially available technology created by Chuck Hull was stereolithography (SLA). This method was based on photopolymerization of liquid resin by ultraviolet light. At the end of 80’s Scott Crump filed a patent for fused deposition modelling (FDM) – a technique which used thermoplastic material for object preparation. In the 90’s Emanuel Sachs - MIT scientist with co-workers patented “Three-dimensional printing techniques” based on joining the selected regions of powder by binding material (3). The most important achievements in 3D printing in pharmaceutical and biomedical applications are presented in Fig. 1.

## How it Works

Among almost 40 years of 3DP history many different techniques were developed and evolved with the technological progress.

The main methods are based on:

- powder solidification,
- liquid solidification,
- extrusion.

**Fig 2:**
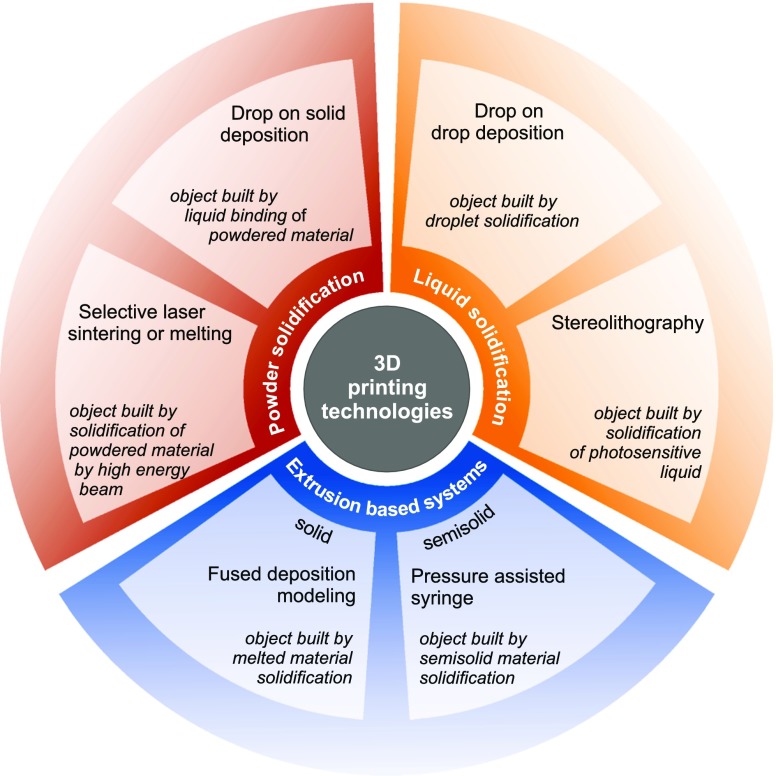
3DP methods applied for drug formulation.

Schematic view of 3DP methods applied for drug formulation is presented in Fig. 2.

**Fig 3:**
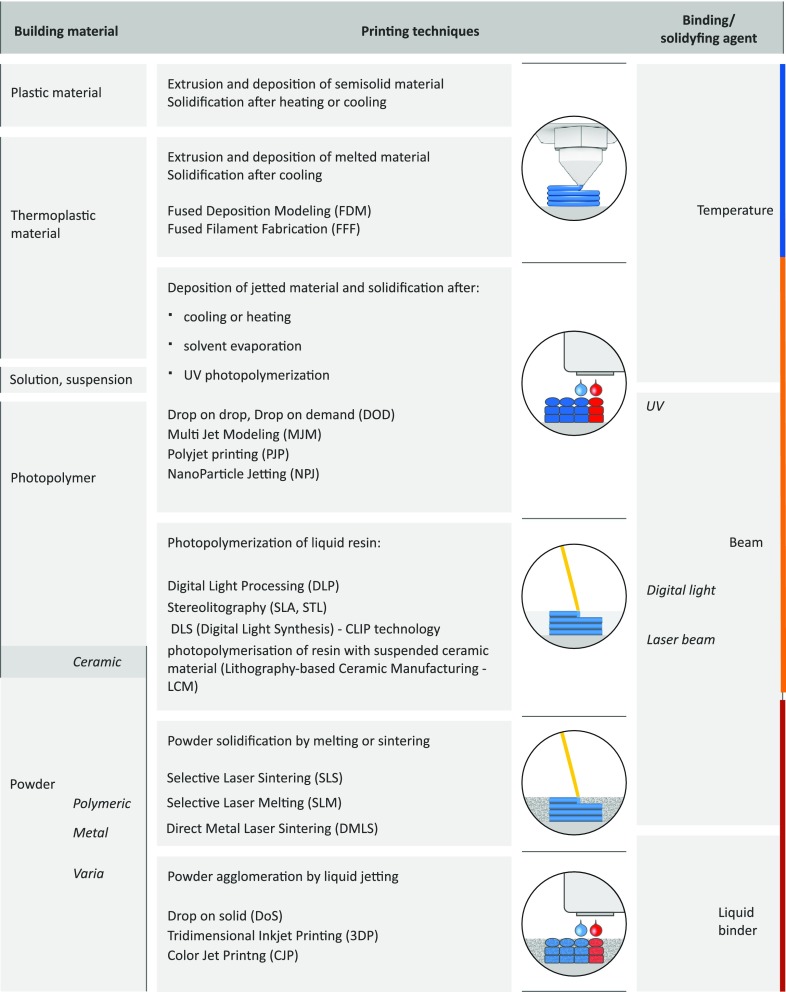
3D printing methods.

Each 3D printer which works according to a different mode requires sufficient material to be solidified and subsequent object fabrication (Fig. 3).

Despite of the diversity of 3DP methods, preparation of 3D-printed object includes several stages:

- the design of 3D object with computer-aided design software and optimization of the geometry according to printer specification,
- the export of 3D model to a common and printer-recognizable file format e.g. STL which includes only 3D geometry in form of each vertex position data or OBJ in which additionally information about polygonal faces or color texture are coded,
- the import of the file to the software and generation of layers which will be printed; the height of the printed layer essentially influences the quality of the printed object as well as printing time,
- the fabrication of the object by subsequent application (or solidification) of the material layers dedicated to the specific printing method.

**Fig. 4:**
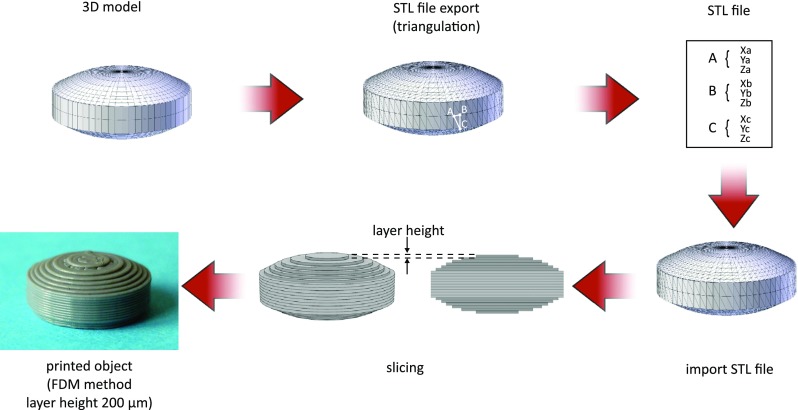
The development of 3D printed object (from ref. (4) with modification).

The development of 3D printed object is illustrated in Fig. 4.

**Fig. 5:**
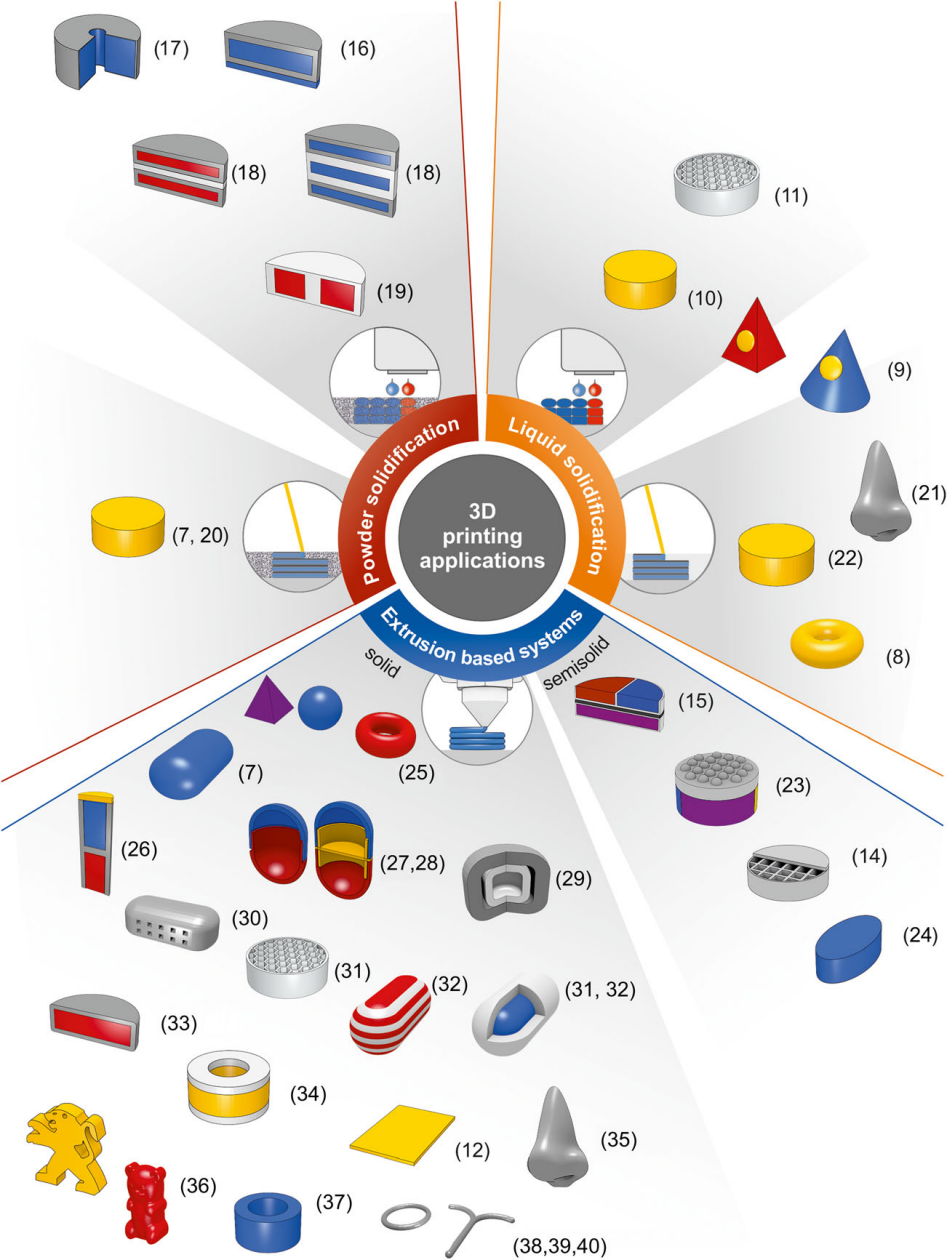
Examples of 3D printed products developed by different 3DP methods with references in brackets.

The 3D printing methods gain an importance in the field of pharmaceutical and medical applications because of the possibility of rapid preparation of tailor-made objects which can be applied in personalized therapy or medicine. The introduction of 3D printing into the pharmaceutical technology particularly aims at the development of patient-centered dosage forms based on structure design. It is still a new research direction with potential to create the targeted release drug delivery systems in freeform geometries. Extensive research are conducted for oral dosage forms because that route of administration still remains the major and the favorite one. Some investigations are also focused on dosage forms for topical administration. The examples of 3D printed products demonstrate the growing interest in drug design by using different 3D printing techniques (Table I, Fig. 5).

**Table I:** Examples of 3D Printed Medicines Prepared with Different 3D Printing Methods

Manufacturing method	Dosage form	API	Excipients	Effect	Ref.
Powder solidifcation					
Drop on solid	Implant	Isoniazide	**Powder:** PLLA **Ink:** Acethone, Ethanole, Water	Sustained release	(5)
	Tablets	Captopril	**Powders:** maltitol, maltodextrin, **Ink:** Water Polyvinylpyrrolidone	Rapidly dispersing tablets	(6)
Selective laser sintering	Orodispersible tablets	Paracetamol	hydroxypropyl methylcellulose vinylpyrrolidone-vinyl acetate copolymer	Orodispersible tablets, fast drug release	(7)
Liquid solidification					
Stereolithography	Tablets	Paracetamol 4-Aminosalicylic acid	Poly(ethylene glycol) diacrylate, Poly(ethylene glycol) 300, diphenyl(2,4,6- trimethylbenzoyl) phosphine oxide	Controlled release	(8)
	Microneedles	Insulin	Dental SG resin Xylitol, Mannitol, Trehalose	Insulin skin delivery	(9)
Drop on drop	Tablets	Ropinirole HCl	Irgacure 2959 Poly(ethylene glycol) diacrylate	Fickian diffusion API release mechanizm	(10)
	Tablets	Fenofibrate	White beeswax	Fickian diffusion API release mechanizm	(11)
Extrusion based methods					
Fused deposition modelling	Orodispersible films	Aripiprazole	Polyvinyl alcohol	Fast disintegration and dissolution	(12)
	Tablets	Theophylline	Hydroxypropyl cellulose, Triacetin, Sodium starch glycolate, Croscarmellose sodium, Crospovidone,	Immediate release	(13)
Extrusion at room temperature	Floating tablets	Dipyridamole	Hydroxypropyl methyl cellulose, Microcrystalline cellulose, Lactose, Polyvinyl pyrrolidone	Sustained release, gastrofloating dosage form	(14)
	Multi-compartment tablet	Nifedypine, Captopril, Glipizide	Polyethylene glycol 6000, Microcrystalline cellulose, Hydroxypropyl methylcelloluse, D-Mannitol, Lactose, Sodium starch glycolate, Croscarmellose sodium, Sodium chloride, Tromethamine	Controlled release	(15)

### From Powder it will Raise

**Fig. 6:**
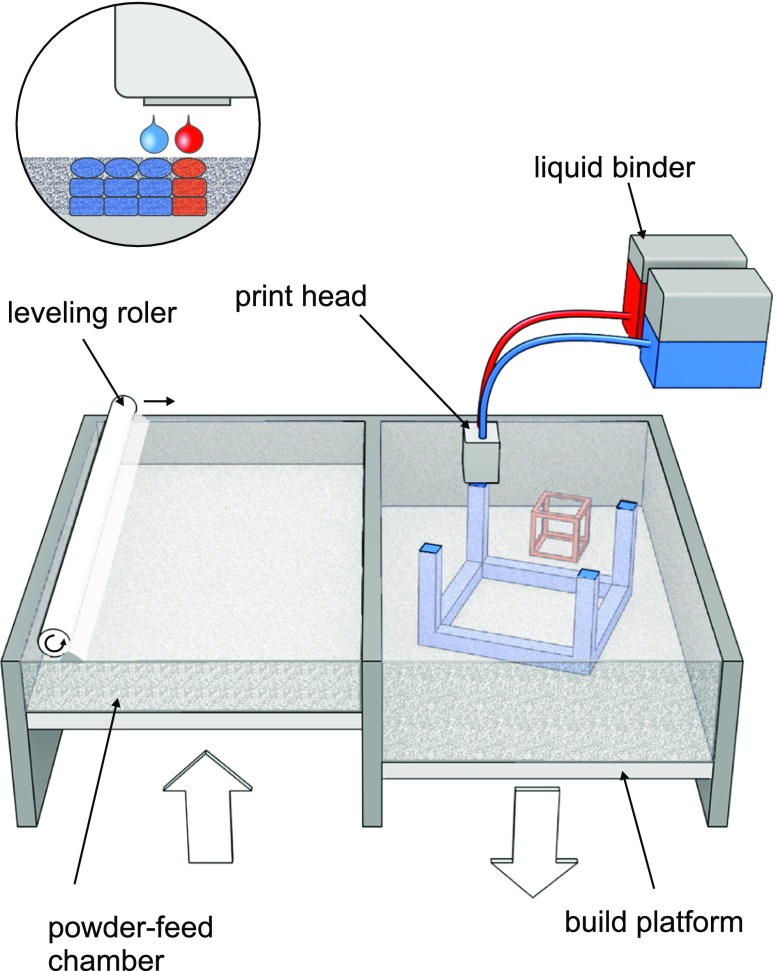
Mechanism of DOS 3D printing (from ref. (4) with modification).

The first 3DP method used in the development of pharmaceutical dosage forms was based on the idea presented in patent entitled “Three-dimensional printing techniques” (3). The printing process mode of action is similar to desktop inkjet printers and is called drop on solid deposition – DOS or powder bed jetting. Droplets of ink sprayed from print head bind the layer of free powder bed while unbound powder particles act as a support material preventing from collapsing of overhang or porous structures. After each step the formed object is lowered and a layer of free powder is applied by roller or powder jetting system and process is proceeded (Fig. 6).

The first printers were equipped with commercially available thermal or piezoelectric print heads that delivered bonding agent. Active pharmaceutical ingredients as well as modifying agents can be either dissolved or dispersed in ink or distributed in powder bed. This method was used due to its similarity to classical formulation processes as wet granulation and the ability to apply the excipients commonly used in the field of pharmaceutical technology, especially in solid dosage form formulations. One of the advantage of this method is possibility of precise location of exact dose of drug or modifying excipients within powdered bed to obtain several compartments with different composition or mode of action. The product quality attributes are influenced by powder and ink properties. The layer height is mainly influenced by particle size and flowability of powder bed as well as cohesion force between particles and part of the printer or powder wettability. Lower layer height and subsequent layer application result in more detailed and precise fabrication of objects with minor mass and dose variations. The ink constituents as solvents, APIs or modifying excipient can change viscosity, droplet size and influence the efficiency of powder binding. The process parameters as printing speed, droplet volume, distance from powder bed also play an important role in the product development and can have an impact on the powder bonding especially between layers in Z axis. They may negatively affect the mechanical strength of the printlets. After printing, additional steps as drying, residual solvents and unbound powder removal should be performed. The DOS method was used in preparation of various solid systems such as implants with levofloxacin (16) or in combination with rifampicine (41), rifampicine and isoniazid (42) exhibiting modified or pulsatile release of APIs. The chlorpheniramine tablets with modified release () and acetaminophen tablets with linear release profiles () were developed as well.

The DOS method is suitable for preparation of easily disintegrating tablet with porous structure. The development in this filed have led to the commercialization of ZipDose^®^ technology by Aprecia^®^ Pharmaceuticals and the first 3D-printed drug Spritam^®^ which was approved in 2015 by FDA (43). The orodispersible tablets which disintegrate within seconds in aqueous solution contain antiepileptic drug levetiracetam. In case of orodispersible tablets high dose of APIs usually results in technological problems in manufacturing and quality control (44). Application of 3DP techniques allowed to prepare fast disintegration tablets with dose up to 1000 mg (45).

Example of Spritam^®^ showed that DOS can be used in a large scale manufacturing of highly porous ODTs. Nevertheless, preparation of multicomponent modified release tables on large production scale with sufficient repeatability of quality attributes can be challenging with regard to more dense structure of this tablets compared to ODT. Variations in porosity resulted from the differences in adhesion between layers can influence the APIs dissolution profile. The application of higher amount of binder prolongs drying time and increases the risk of limited removal of residual solvent.

**Fig. 7:**
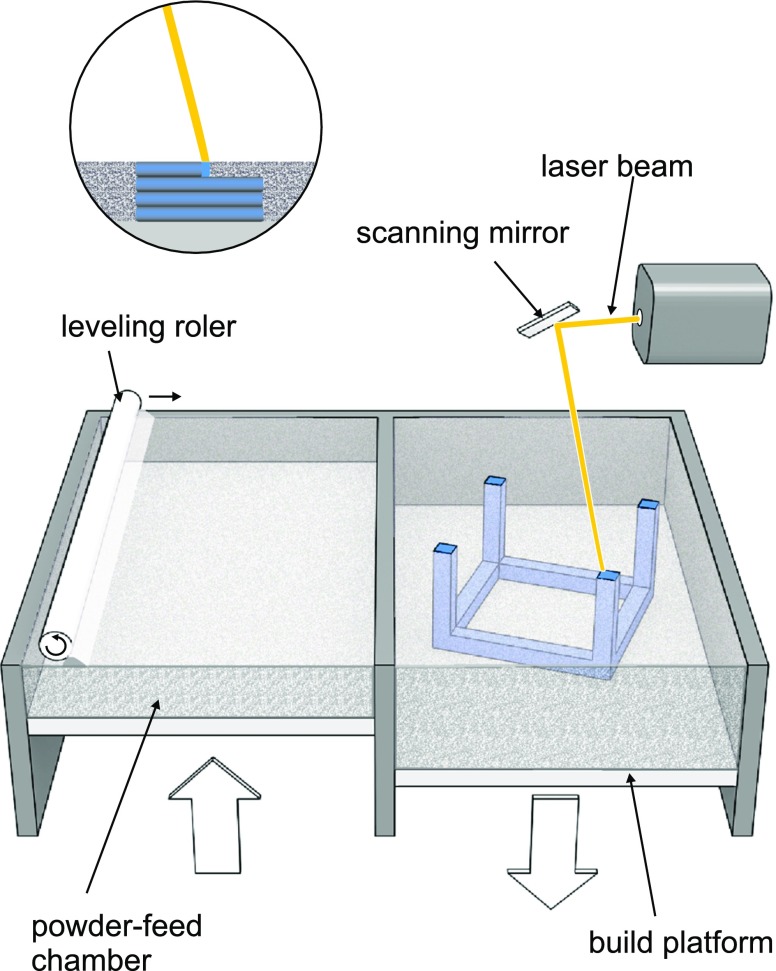
SLS and SLM technology.

A powder can be also solidified by applying the beam of high energy. The basic construction assumptions of selective laser sintering or selective laser melting (SLM) are similar to DOS method. Powdered bed is transferred from one compartment to another by levelling system and layers are formed by sintering (heating just below melting temperature) or melting the polymeric or metallic powdered bed by laser beam (Fig. 7).

In this process the high energy beam is created usually by laser and applied power varies depending on powder properties. While 5 W laser is sufficient in case of polyamide, even 1 kW laser is required for metal melting. Powder bed must be heated before printing in the printer chamber to the temperature adequate to the process parameters. After printing the fabricated objects are imbedded in powder and the bed should be slowly cooled down to avoid stress. These steps are time consuming and depends on printer chamber volume. Another drawback is the risk of API decomposition after exposure to laser beam. Despite all the disadvantages, the SLS method is under consideration as a way of solid dosage form formulation. Fina et al. () used SLS for preparation of paracetamol tablet with Kollicoat^®^ IR or Eudragit^®^ L100–55. Additionally, Candurin^®^ gold sheen was added to aid the sintering process. The produced printlets were characterized by good mechanical properties - low friability in the range of 0.02–0.56% and high crushing strength - from 284 N to over 485 N. The drug content was equal to the theoretical value. The pH-independent release characteristics with the release rate dependent on the drug content were observed for Kollicoat^®^-based formulations whereas Eudragit®-based formulations showed pH-dependent and modified-release profiles not correlated with the drug loading. The application of another polymers i.e. hydroxypropyl methylcellulose or Kollidon^®^ VA 64 and optimization of printing parameters influenced printlets characteristics. Increased laser scanner speed resulted in more porous structure and decreased mechanical strength. In case of Kollidon^®^ VA 64 printlets showed fast dissolution characteristics (over 90% after 5 min) and rapid disintegration time - 4 s (7).

SLS technology can be a promising method to obtain porous, rapid disintegrating as well as modified release dosage forms without binding agent. The decomposition of APIs may be induced by high energy beam especially during preparation of more dense form. The stability issues and production time seem to be important challenges in SLS application. Sintering speed influencing the porosity of the printlets and effectiveness of printing process can be increased by multiplication of laser beams like in metal sintering 3D printers.

### Turning Liquid into Solid

**Fig. 8:**
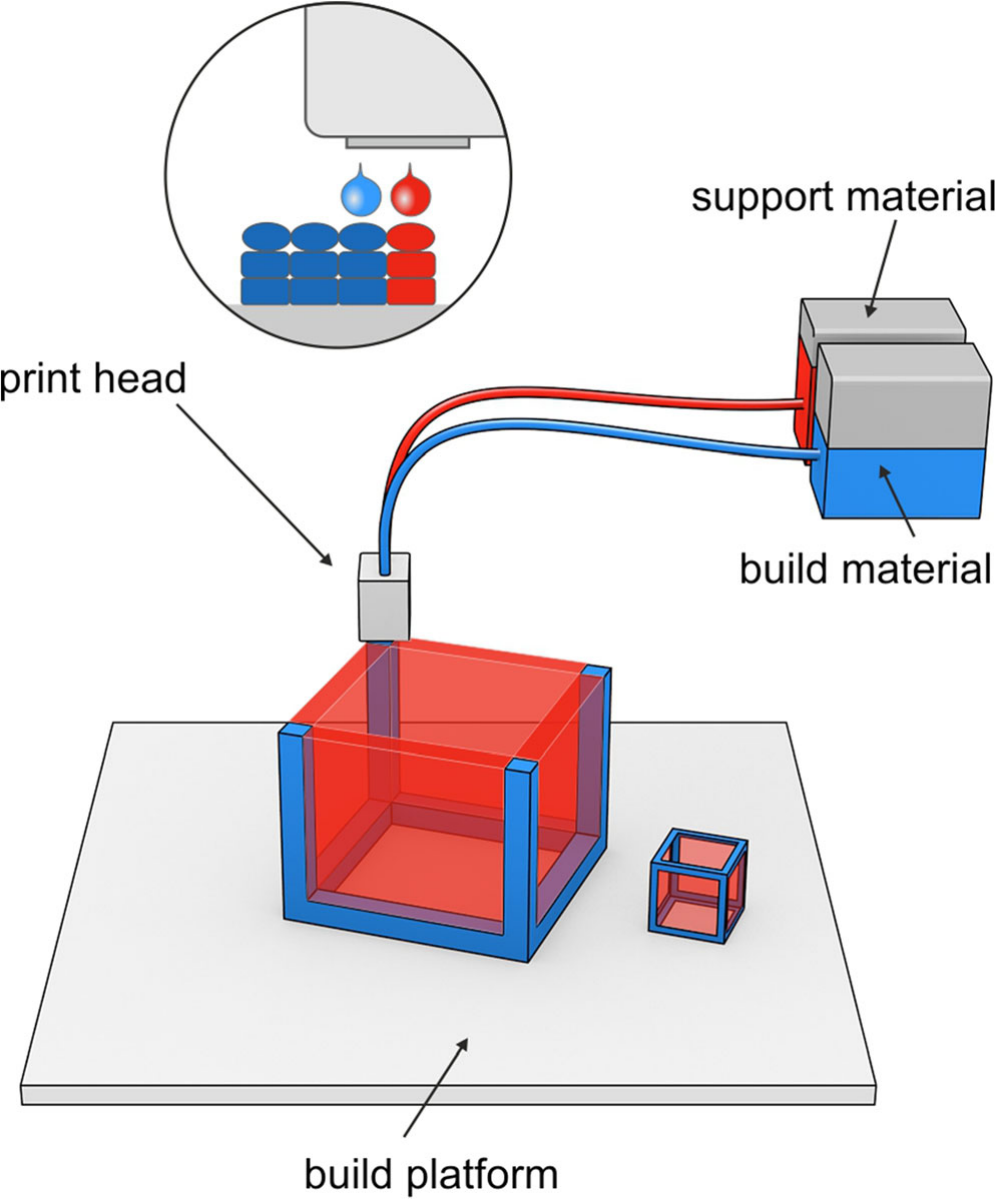
DOD deposition technology.

The idea of object manufacturing by solidification of liquid is similar to powder solidification technique. Droplets of “ink” sprayed from the nozzle are deposited on the thin layers and cured by cooling air or in presence of high energy light. Due to the absence of powdered bed the drop on drop (DOD) or Polyjet technology requires the use of additional material to create support for overhang geometries. The print head moves along X and Y axes during printing, the print platform bed is lowered along Z axis by the height of the layer after the deposition of each layer of the material (Fig. 8). Different technical solutions applied in this technique resulted in possibility of multi-material full-color printing.

The quality of printed product as well as curing method depend on properties of spraying material. The first materials used in this techniques were waxes. In pharmaceutical technology they were used in modified release dosage form formulation and at present time also found application in 3DP technology. Molten wax was sprayed on build platform in heated chamber to prevent rapid solidification. Matrix tablets containing beeswax and fenofibrate were prepared by Kyobula et al. (11) by means of 3D material jetting of molten wax and the influence of bespoke geometries (honeycomb architecture with different infill ratio) on dissolution profile was revealed. The cell diameter of the honeycomb geometry and material wettability were found to affect the dissolution profile from tablets of constant weight. The amount of released drug increased with honeycomb diameter and surface in case of middle size honeycomb channels. For the smallest dimension of honeycomb, penetration of dissolution medium was insufficient whereas surface of tablets with widest honeycomb was smaller than middle size structure what resulted in decreasing of drug dissolution in both cases. This phenomena can allow the production of personalized medicines when applied with combination of different materials of various geometry.

Photosensitive polymers can be also used in this techniques and solidification of the layers is achieved by UV light. To assess the possibility of photopolymer resin application in 3D printing of drugs, tablets with ropinirole HCl, poly(ethylene glycol) diacrylate (PEGDA) and Irgacure 2959 as photoinitiator were prepared in an environment with low content of oxygen which can inhibit the curing transformations. Average printing time of tablets of 14 mg mass having 5.02 mm in diameter and 0.72 mm in height was ca. 4 min. Crosslinking of UV-sensitive material resulted in formation of amorphous solid dispersion of ropinirole HCl exhibiting sustained release of API from tablet up to 6 h (10). In this technique post-process removal of unbounded polymer and photoinitiator is important step. Toxicity of the components should be considered when this process is applied in drug formulation.

This first attempts of DOD method application in drug formulation field revealed that tested matrix materials are suitable in prolonged release dosage forms fabrication. Challenging process of adaptation of a wide range of polymers needs to be evaluated. The modification in wax matrix composition or application of hydrophilic polymers may have a great impact on dissolution behavior. In case of solidification method based on UV light, possibility of APIs decomposition and stability matters should be taken into account. The post-processing of dosage form as well as the toxicity and removal of unbounded monomer and photoinitiator should be also considered when this process is applied in drug formulation.

**Fig. 9:**
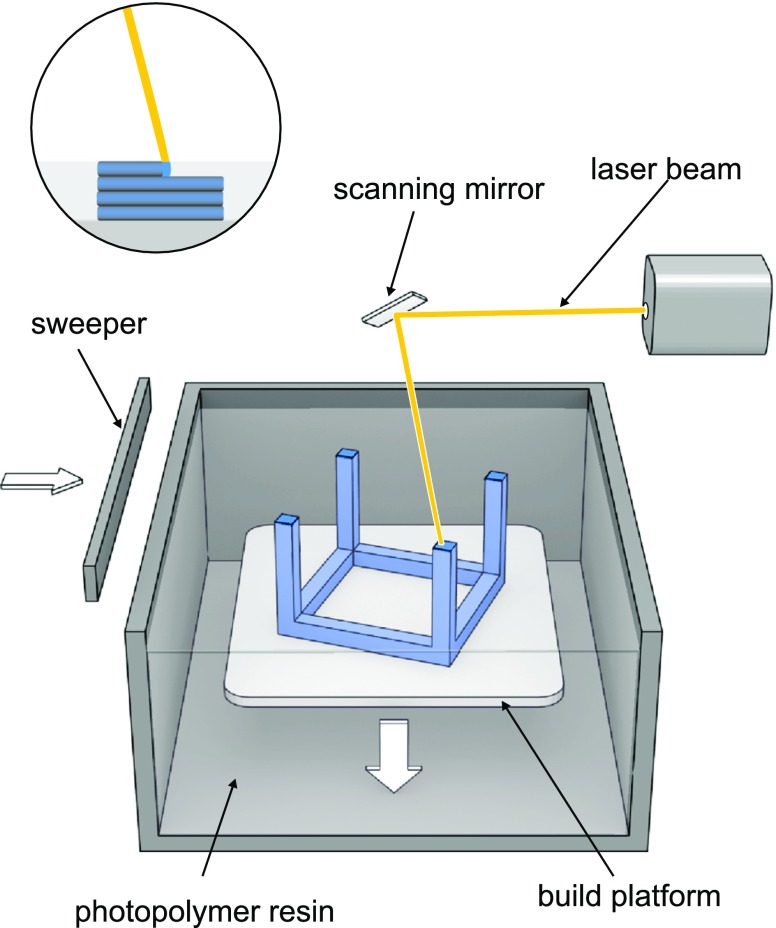
SLA technology.

Photosensitive liquid polymers are also used in stereolithography. The object is built by solidification of subsequent layers of resin in the presence of high energy light e.g. UV laser beam or light from projector (digital light projector - DLP). Laser beam guided by scanner mirrors precisely “draws” layers of the object on build platform by cross-linking of the photopolymer. In DLP techniques projector displays image of the whole layer. Printed object is bound to the build platform which is immersed in the photopolymer and layer is tracing on the surface of the resin. Subsequently, platform descends a distance equal to the thickness of a single layer and sweeper recoats object surface (Fig. 9).

In inverted SLA, source of light is situated under transparent bottom of a resin tank which is coated with a non-sticking material. Build platform is lowered from above to the distance equal to layer height and object layer is solidified. In the next step object is raised and sweeper cleans tank bottom. This mode of action allows to efficiently use the resin and smoother layer surface. Wang (8) used polyethylene glycol diacrylate as a resin and diphenyl(2,4,6-trimethylbenzoyl)phosphine oxide (DPPO) as photoinitiator to evaluate the suitability of stereolithography (SLA) to fabricate 4-aminosalicylic acid and paracetamol-loaded tablets with modified-release characteristics. Tablets containing different amount of polyethylene glycol 300 (PEG 300) were successfully printed and increasing PEG300 content revealed enhancement of drug release within 10 h dissolution studies. Martinez et al. () used also PEGDA as polymer but try to evaluate possibility of application of the nontoxic photoinitiator – riboflavin to obtain ibuprofen containing hydrogels with different water content. Hydrogels containing up to 30% w/w of water, and 10% w/w of ibuprofen, were successfully printed. Dissolution profiles showed that drug release was faster from hydrogels with higher water content.

The challenges in SLA application are similar to photopolymerization DOD method and are related to stability and safety issues. Additionally, in SLA method the objects are printed with solid infill and modification of porosity can be obtained only by changing the geometry of the tablet.

### Principles of Extrusion-Based Methods

Hot melt extrusion (HME) as well as extrusion of semisolid materials are well established processes in the field of pharmaceutical technology. Increasing popularity of printing methods based on this technical solution is related to progressive availability of compact size and relatively inexpensive equipment. Essentially two modes of printing method can be distinguished:

- extrusion of semisolid, or semi-molten materials (gels, pastes) at room or elevated temperature,
- extrusion of molten thermoplastic rod-shape material (filament).

**Fig. 10:**
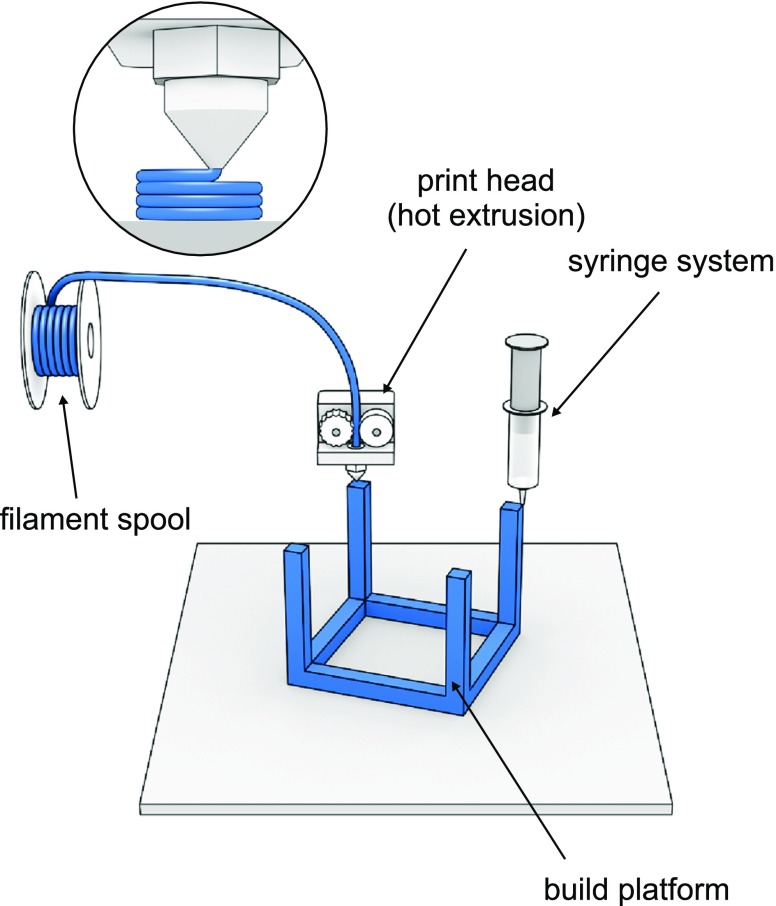
Extrusion-based techniques.

In both modes the material is extruded from the nozzle and spread in subsequent layers on the surface of build platform (Fig. 10). Defined dimension of printed path is created by the distance of print head to the build plate and influenced by the nozzle orifice diameter. These two parameters and print speed affect the quality of printed object. Another layer is applied when print head or print platform moves along Z axis at the distance of layer height. The 3D printers technological solution are depending on printing material.

#### Filament – The Major Challenge

The fused deposition modelling is based on extrusion of molten thermoplastic material. Dimensions of filaments in the range of 1.75 mm and 2.85–3 mm are adopted to standard commercially available print heads. Standard filaments are made of thermoplastic polymers such as acrylonitrile butadiene styrene (ABS), poly(lactic acid) (PLA), high impact polystyrene (HIPS), polyethylene terephthalate glycol-modified (PET-G), nylon. There are some commercially available high-quality filaments processed from medically grade polymers such as PLA, PVA, nevertheless the filaments prepared from pharmaceutical grade polymers and containing APIs are not commercially available so far. To prepare drug-loaded filaments the thermal stability of impregnated API must be considered. In FDM process the filament guided by gears is moved towards heat end where it is melted and pushed forward through the nozzle orifice by unmelted filament. The diameter of the nozzle orifice varies from 0.2 to 0.4 mm, and has an impact on resolution of printed object. Usually the printed path width is equal to the orifice diameter and the height of the path is a half of its width, however the final settlements can vary depending on the printed material and printer settings. The paths are organized in layers and formed into the object which resolution depends on layer height. The mechanical parameters of the printed object are related to number of outlines which build the external wall of the object and infill parameters ratio and type (e.g. honeycomb or rectilinear).

The filament quality attributes like constant dimension, elasticity, stiffness, homogenous drug distribution are of the key importance in the development of printed dosage form by using FDM method. Nowadays, HME is a main way to obtain good-quality filaments containing APIs, however the first attempts of FDM method application were based on ready-to-use, commercially available filaments for 3D printers. Model drugs were incorporated into the filaments by swelling filament in volatile solvent solution of API and drying. This approach allowed to prepare first 3D-printed dosage form by FDM method. Nevertheless, the drug content in the filaments prepared in this way ranged from 0.063 to 0.3% for ethanolic solutions (46) and 1.9% for methanolic soaking solutions (47) what was a limitation in higher dosage form fabrication. The simplest solution of this problem was a re-extrusion of shredded or milled commercially available filament with APIs by HME. This process is designed to result in filaments with higher drug content and mechanical properties suitable for 3DP (46,48). Li Q. et al. (14) used this method for preparation of PVA-based filament containing 5% of glipizide. The development of dosage form formulation requires high quality pharmaceutical grade excipients which can be used in extrusion process. The excipients that meet the extrusion requirements should be characterized by proper glass transition temperature (T_g_), i.e. lower than drug decomposition temperature, matrix forming capability and good rheological properties. Therefore, the printability of raw materials requires a deep understanding of the printing process, taking into account physical and chemical characteristic changes during its selection. As a result of HME process filaments with sufficient mechanical attributes should be obtained because during printing process filaments are bended and compressed between feeding gear and driving gear. Very brittle filaments can be broken by the gears whereas too soft filaments may be squeezed aside by the feeding gear. Zhang et al. tested different polymers: HPC, HPMC, EC, Soluplus^®^, Eudragit^®^ L100 and combination of thereof as filament formers in HME process. The 3-point bend test was used to assess the mechanical properties of the prepared filaments and the results were compared with data obtained for PLA commercially available filaments. This approach allows to recognize border parameters and indicates which filament can be printable (49). The properties of starting materials for filament fabrication have a great impact on their printability, therefore the selection of appropriate HME-based matrices is required (50). Different matrix polymers, plasticizers and fillers were used to improve printability while lubricants were essentially used to reduce friction between filament and walls of the printing extruder (51). As a result of multicomponent formulation, high drug-load filaments with good mechanical properties can be obtained. Goyanes et al. (52) prepared filaments containing 50% of paracetamol based on HPMCAS with addition of methylparaben as plasticizer and magnesium stearate as lubricant. Drug loading in filaments depends on properties of APIs and excipients as well as the extruder construction and process parameters. High theophylline and metformine-loaded filaments were obtained by Verstraete et al. (). Different grades of hydrophilic and hydrophobic thermoplastic polyurethane were used as a matrix formers and filament with 60% of APIs was obtained. High content of crystalline drug resulted in rough surface of the filament which was reduced by milling of APIs prior to the extrusion process. After milling filament with consistent diameter, smooth surface morphology and good mechanical properties were obtained. The predefine printing parameters were found to influence printlets behavior. The infill ratio which varied from 25 to 100% had an impact on dissolution behavior of API. The decrease in drug dissolution with increased infill ratio was correlated with decreasing porosity. However, layer height from 200 to 400 μm as well as outline counts from 1 to 3 of printed tablets had no influence on drug dissolution profiles. The influence of infill ratio on dissolution profiles were reported in several publications (53). In case of paracetamol 5 and 50%-loaded tablets the influence of infill on dissolution profile was also observed. Additionally, the application of different grades of entering coating polymer (HPMCAS) as matrix forming polymer resulted in delayed dissolution profile characteristics. The drug amount released after 2 h in acidic medium did not exceed 10%. By combination of infill ratio and different grades of polymers the modified release dosage forms with different dissolution profiles were prepared (52).

#### Geometry Matters

The printlets shape also influences the drug dissolution rate. Paracetamol tablet of different shapes with constant surface were proposed by Goyanes et al. (). The fastest dissolution rates were obtained for pyramid shape formulations which has the biggest surface area/volume ratio whereas the cylindrical or spherical shapes with smallest surface area/volume ratio were characterized by the slowest dissolution ratio. Modification of tablet geometry by addition of the extra channels was also proposed to accelerate hydrochlorothiazide dissolution. Squared, in cross section, channels with diameter ranging from 0.2 to 1.0 mm were imbedded during designing stage. The dissolution data revealed that channel of size ≥0.6 mm essentially accelerated the drug release which meets pharmacopoeial criterion for immediate release products (). Another innovative approach of tablets was proposed by Arafat et al. Tablets were composed of bridged 9 units with spacing gaps. The different block and gaps size influenced the disintegration and dissolution time. The designed approach is an interesting alternative to disintegrants application to accelerate tablet disintegration (13).

#### Two Heads Are Better than One

In dual head extrusion process, the print head is equipped with two separate stepper motors and heating compartment which allows to carry out the process with two materials characterized by different melting temperature. Okwuosa et al. (51) prepared the delayed release tablets with theophylline loaded core, based on PVP polymer, printed by one extruder and outer complementary shell of Eudragit^®^ L with increasing thicknesses from 0.17 to 0.87 mm which was prepared by second printing extruder. The shell thickness which was required to achieve sufficient core protection in the acidic medium was ≥0.52 mm.

Dual head solution can be also used to fabricate dual layer modified release tablets. Li et al. () proposed concept of DuoTablet – dosage form built with two (inner and outer) compartments. The core of the tablet as well as the outer shell were printed with glipizide-loaded PVA filament containing different ratio of API, 4.8 and 2.2%, respectively. The dissolution studies revealed modifications of drug release profiles in comparison to the tablets printed with one placebo compartment. This approach can be a promising method of controlled drug delivery system preparation.

Dual head printer was also used in fabrication of another dosage form: dual-compartmental dosage unit (dcDU) which is a two compartment tube printed with insoluble PLA. During printing of the first compartment of the tube the process was paused and tube was manually filed with rifampicine or isoniazid loaded filaments. Printing process was continued and paused when the second compartment was finished to manually load the filament containing the other drug. Sealing PVA cap was printed on the one side of the tube at the end of the printing. The construction of the unit provides the separation of incompatible APIs in single dosage form and differentiation of dissolution profiles which shows lag time as a result of cap seal (). Placebo structures influencing the dissolution profile were also proposed by Maroni et al. (). Commercially available PVA filament as well as filaments prepared by HME with pharmaceutical grade polymers (Kollicoat^®^ IR, HPMC, HPMCAS) were used to fabricate three parts of dual compartment capsular device which two hollow parts could be filled extemporaneously with different content and connected with the joining part. The polymers used for the device preparation and wall thickness of the hollow parts (600 or 1200 μm) resulted in modulation of bimodal released profile.

One of the advantage of FDM method is the possibility of API amorphization during printing or HME filament preparation. API can be melted and/or dissolved in polymer matrix according to the polymer properties and process parameters and this phenomena may improve drug dissolution rate (54). Aripiprazole-containing orodispersible films (ODF) prepared by FDM were compared with casted films. Drug in casted films was crystalline whereas in printed films was fully amorphous on account of two step hot melt extrusion (filament fabrication and 3D printing) what positively improved the dissolution profile of aripiprazole from 3D-printed films. This study showed that FDM is a technique that can be used as alternative to commonly used for the preparation of ODFs (12).

Extrusion-based methods are very promising. They provide easy modification of the infill structure of the printlets, whereas other techniques (SLA, SLS) require new 3D models. However, during filament aging undesirable interactions between filament components and variation in moisture content can lead to changes in physical stability e.g. recrystallization of API. This can impact mechanical properties of filament and results in increased fragility or some shifts in melting temperature what can negatively affect the properties of printed dosage form.

#### Extrusion of Semisolids

In case of printing of semisolid, or semi-molten materials (gels, pastes) at room or elevated temperature by extrusion process, some changes have been implemented in print head construction in comparison with FDM. The mass is extruded through orifice by compressed air pressure, syringe plunger or screw. This systems allow to fabricate high drug load dosage forms, nevertheless after printing the drying step is required what can influence the product integrity. Immediate release paracetamol tablets with 80% of drug load were prepared with pharmaceutical grade excipients that comply with pharmacopoeial standards (). The systems with modified release were prepared as well. Li Q et al. (14) developed gastro-floating tablets with dipyridamole to prolong the gastric residence time. *In vitro* buoyancy study revealed that 30 and 50% of infilling rate formulations floated up to 12 h. Multi-syringe printing system allows to prepare “polypill” as multi-active solid dosage form containing 3 or 5 APIs released with different kinetic characteristics (15,).

## Patient-Centric Therapy

Due to the possibility of various materials utilization, 3D printing methods have wide range of applications in medicine e.g. to build spatial systems used in tissue engineering as well as in pharmacy to prepare such dosage forms as tablets (55), capsules (), implants (,^56^) or orodispersible films (12). As already mentioned, tablets are the most frequently produced dosage forms. While they can be manufactured in several geometries, very few doses for one API are available on the industrial scale (15,57).

The idea of more individualized pharmacotherapy has been developed for many years, however its meaning has never been greater as at present time. The need for developing personalized medicine by rational use of drugs by the patients in right dose is a subject of intensive discussion because heterogeneous nature of diseases is the source of difficulties in therapeutic intervention. The therapy failures or limitations of therapeutic effects are some of the reasons to modify the dosage form as well as dose of the active substance especially for individual age groups. The appropriate dosage forms need to be selected considering not only physicochemical properties but particularly target population and treated disease. The development of pharmaceutical product for pediatric and geriatric population is especially recommended due to the diverse needs and characteristic of each patient group. Due to the dose flexibility and difference in swallowing, tablets subdivision into two or even four parts is a common practice in health care. The problems with scored tablets has been reported in the literature. Unequally breaking and loss of mass after division finally could lead to over- or under dosing (58). Therefore, the implementation of three-dimensional printing may became extremely useful in the development of personalized therapy.

The 3D printing allows to individualize medicine to the patient body weight and lifestyle by dose and dosage form adjustment, e.g. orodispersible tablets instead of conventional tablets for active or noncompliant patients. The simplicity of preparation of the medicines with different doses is caused by the scalability of the designed objects, thus the dose can be controlled by calculated material consumption during resizing of the printed object already at the design stage. This manufacturing method seems to be especially beneficial in the production of orphan drugs made for small groups of patients. Relatively low cost of production of dosage forms with different doses is one of the major advantage in term of short series of medicinal product (15,57).

The 3D printing brings unprecedented opportunities for the development and preparation of personalized medicines at pharmaceutic or industrial scale. The introduction of 3D printers to the hospitals and community pharmacies would raise the pharmaceutical compounding to entirely new level. Especially in case of pediatric patients not only the dose of the active ingredient is important but also the quantity of the administered medicines, their shape, color and taste play a huge role in therapy (59). Moreover, in some 3D printing methods, like fused deposition modelling, where APIs are incorporated into polymer matrix, taste masking is achievable without any further processing such as film coating. Using this 3D printing method Scoutaris et al. () printed taste-masked dosage forms in the shape of Starmix^®^ Haribo jelly beans with indomethacin with good reproducibility, accuracy, content uniformity, and fast dissolution of the API. The size and the shape of the tablet affect patient acceptability, especially in term of swallowing difficulties. Tablet shape is also of crucial importance to the elderly patients not only due to the swallowing problems but also in accordance with the manipulating problems. Goyanes et al. (60) investigated the influence of different tablet shapes i.e. sphere, torus, disc, capsule and tilted diamond shape on the patients feelings in term of the ease of swallowing and handling. It was found that the easiest to swallow were doughnut shape i.e. torus tablets. The tablets of conventional shape were also defined as acceptable because of their similarity to standard dosage forms. In the previous studies the same team stated that this shape differences slightly influence the dissolution behavior so there should be a freedom of choosing the geometry for individual patients (). The effect of geometry and internal structure on the printed dosage forms properties is a separate issue and it was discussed in the FDM section of this article.

The 3D printing gives the opportunity for producing tablets with more than one active substance characterized by different properties and with different dissolution profiles. Thus, it can result in the reduction of the amount of used products by formulating complex medicines (61). By using of 3D printing technology the precise control over dissolution behavior can be achieved mainly by applying selected soluble or non-soluble excipients but also by designing the specified geometry and internal structure of the printed dosage forms (62). Nevertheless, this opportunity should only be used by healthcare professionals because it requires the knowledge about pharmacokinetics of the active substance and health condition of the patient and it could be applied in hospital pharmacies.

Although the solid oral dosage forms have been the most widely studied, the 3D printing was also utilized to fabricate transdermal drug delivery system. Luzuriaga et al. (63) printed microneedles with fluorescein. Additional etching step was necessary to achieve satisfactory microneedles shape. In the latest research on microneedles stereolithography was applied to obtain needles with appropriate shape and size and inkjet print was carried out to coat the microneedles with insulin formulation. The authors obtained rapid *in vitro* dissolution of insulin but the most important was the possibility to produce microneedle patches in a simple way (9). The use of microneedles can assure painless transdermal delivery of drugs and vaccines, however the manufacturing technologies are still difficult to apply in pharmaceutical industry (64). Nevertheless, 3D printing is promising method of fabrication of different microstructures.

The additive manufacturing methods were used to produce other topical delivery systems. Vaginal rings for controlled topical delivery of progesterone were successfully manufactured by J. Fu et al. (39) by fused deposition modelling. The authors emphasized that particular drug delivery device is strongly needed to be personalized due to the different needs of the patients and a lack in the market of such personalized vaginal delivery systems.

### From Patient Home to Production Scale

The 3D printing of the medicines in patient facilities is intensively discussed in the scientific communities but additive manufacturing techniques are still rather new for the society and vision that patients will print their medicines in their own houses is rather faraway perspective. The main obstacle to the implementation of this method to the patients is drug quality and safety issue. Patients or their medical care have to be thoroughly trained how to use printer and how to recognize quality defects that may appear in their self-printed drug. However, this approach can be beneficial for the patients as a higher involvement in the treatment process which was found to be clinically beneficial (65). On the other hand, it can also lead to some disadvantages like loss of control over adverse effects when formulating polypills with many active substances within one medication.

As it was mentioned before 3D printing can be relatively easy introduced into the pharmaceutical compounding in the pharmacies. Fused deposition modelling seems to have the nearest way because having good quality of API loaded filament and database with objects to print, medically-educated staff i.e. pharmacist can print the final dosage form with defined architecture and dose of the active substance (65). The manufacturing of API-loaded filaments is not a big challenge for pharmaceutical industry because hot melt extrusion, which is the basic method of filament preparation, is well established in the pharmaceutical manufacturing. Printable filaments have been obtained so far from number of pharmaceutical grade polymers, i.e. cellulose derivatives, methacrylic acid copolymers, poly(ethylene oxide), poly(vinyl alcohol), poly(vinyl alcohol)-poly(ethylene glycol) graft copolymer, poly(vinyl caprolactam)-poly(vinyl acetate)-poly(ethylene glycol) graft copolymer, poly(ethylene glycols), ethylene vinyl acetate and others (12,50, 38). One of the advantages of this 3D printing method is the possibility to produce filaments with incorporated API in crystalline state, which can be disordered during extemporaneous 3D printing. This approach can overcome the stability issue of amorphous drugs which are frequently formed during 3D printing because of the mechanism of the 3D printing process which is often based on melting of API with polymer or fast evaporation of the solvent from drug solution (66).

The introduction of 3D printing into the pharmaceutical industry as a standard manufacturing method is still far away from present mainly because of the lack of ready-made production equipment. However, trends have been set by the first registered and marketed 3D-printed drug production method developed by Aprecia^®^ Pharmaceuticals. In this industrial scale pharmaceutical printing technology the formation of tablets is carried out by repeated cycles of building layers by the one feeding, liquid applying system on a conveyor (67). However, this is not the only possibility, other potential method could be the use of a number of print heads, and in case of powder bed bonding powder feeding zones, strictly corresponding to the number of layers of the tablet on a moving platform. In conventional 3D printers single nozzle is used to print whole object. Taking into account that one tablet is printed often for more than 1 min the effectiveness of such method is very limited. In this methods object has to be removed after printing and afterwards the printing process can be repeated. The printing on a conveyor is faster and eliminates the need for moving the nozzle in the Z axis because successive print heads can be fixed higher by layer height. One thing is certain, without developing equipment designed especially for pharmaceutical applications 3D printing will be still in the research phase and will not get to the pharmacotherapy. Tranfield et al. (68) gives directions what should characterize the ideal pharmaceutical 3D printer and emphasizes that only the collaborative work of 3D printer manufacturers, scientists, excipient suppliers and pharmaceutical regulators can result in developing the ideal 3D printer.

## Letter of Law

Three-dimensional printing is rapidly growing technology that is characterized by the high potential from the pharmaceutical point of view Although it is still at the initial stage of considerations, many attempts have been made to scale up this technology and 3DP has become a successful method especially in personalized medicine. However, comprehensive research is still necessary to make the 3DP techniques industrially feasible for dosage form formulation. Nowadays, only one FDA-approved product is on the market. The printable products must comply with the current manufacturing and control standards for the medical products and devices. Current research shows both benefits of 3D printing technology and its limitations. Therefore, due to the large number of the factors affecting the quality of computationally designed dosage forms and safety of their use, the appropriate regulatory requirements are very desirable. Nowadays, there are no valid regulations concerning design, manufacturing process and quality testing considerations. There is a strong need to make some regulations for this particular group of manufacturing methods. Food and Drug Administration guidance released in December 2017, entitled Technical Considerations for Additive Manufactured Medical Devices gives the number of guidelines considering the main aspects from software and hardware requirements, quality control up to process validation procedures (69). As it is highlighted in the FDA document due to variability of additive manufacturing methods there is no possibility to give one universal set of guidelines for all of the 3D printing methods. For example, processes such as SLS and FDM cannot be compared because of different starting materials, i.e. powder for SLS and filament for FDM, different process parameters, i.e. laser beam energy, powder bed temperature etc. for SLS and extrusion and printing platform temperature for FDM technology. There are also some differences in post-processing in the printing methods: some of them, i.e. selective laser sintering, stereolithography or drop on solid methods require removing of manufacturing material residues while the other methods like fused deposition modelling or drop on drop method do not. It seems that every single printing method needs separate regulatory. It has to be underlined that there are still no regulations on medicinal products which are often more demanding than the requirements for medical devices. All of mentioned issues for devices should be considered in case of medicines as well. Moreover, the presence of API makes much more aspects have to be taken into account such as possible incompatibilities, active substance stability during printing process etc. This is of crucial importance because fused deposition modeling method utilizes high temperature twice, i.e. at the filament extrusion stage and even higher temperature during printing. The possibility of active substance amorphization and its stability as well as the influence on the product properties have to be tested and those should be specified in relevant guidelines.

It is expected that 3D printing will enter the pharmacotherapy sooner or later in one of the described options. If so, there is also a strong need to train the specialists in pharmaceutical 3D printing. Both physicians and pharmacists teaching program should be extended by the topics concerned with additive manufacturing methods and their application in the patients treatment.

## (Bio)Medical Applications

**Fig. 11:**
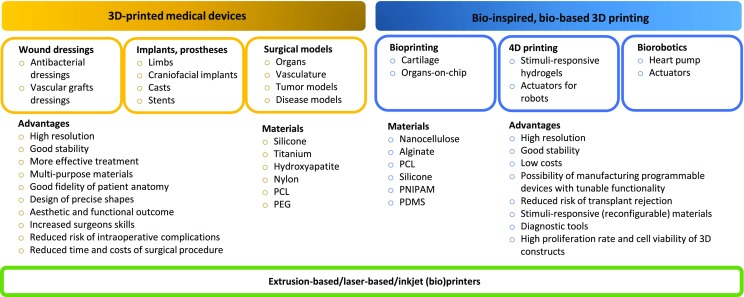
Biomedical applications of 3D printing.

The impact of additive manufacturing on biomedical field has increased rapidly since the 3D printing was developed in the early 80′. It is due to the fact that the technique enables formation of individually developed materials of customized architecture and functionalities. It evolved into powerful tool for biomedical engineering providing formation of manufacturing implants that correspond to patient-specific anatomy, phantoms for education and surgical planning and disease models (Fig. 11). Moreover, additive manufacturing learns from the nature what leads to formation of smart materials or devices.

### Wound Dressing

Growing demand for tailor-made functionalized materials has become a driving force for the development of additively manufactured structures. Nanotechnological approaches address many of challenges faced by modern medicine, however the safety of their use is still under intensive investigation. Although such approaches provide application of antibacterial nanoparticles and carriers of factors improving the wound healing, they are difficult for industrial application. It opens a new challenging field for additive manufacturing as a technique sufficient to produce personalized and safe materials of complex architecture and functionalities.

The manufacturing of patient-specific antimicrobial wound dressings made from polycaprolactone (PCL) with incorporated zinc, copper and silver was described by Muwaffak (). The metal-homogeneously-loaded filaments were obtained using hot melt extrusion and 3D models of nose and ears were printed. The wound dressings exhibited prolonged release of the different metals and bactericidal properties. The anatomically adaptable dressings were concluded to be more affordable than conventional flat dressings.

The 3D-printed hybrid scaffold based on poly(ethylene glycol) (PEG) and homogenized pericardium matrix was developed in order to promote wound healing in vascular grafts that can support the replacement of injured vessel wound after surgical reconstruction (70). The incorporation of homogenized pericardium into PEG matrix affects the modulus of the scaffold as well as reduces the inflammatory signal of macrophages. The obtained biomaterial was described as very promising in terms of vascular graft development and opening a new field in congenital heart defect reconstruction.

### Implants and Prostheses

The fabrication of implants and prostheses by additive manufacturing has recently revolutionized the area of developing of medical devices fulfilling the growing demand for personalized therapy. The 3D printing enables preparation of tailor-made products that meet individual needs resulted from specific patient anatomy and pathology. Moreover, it provides the development of structures of site-specific mechanical and physical properties as well as spatial and temporal control of bioactive components.

The custom prosthetics attachments and devices enable to restore mobility and functions as well as normal appearance lost by deformations or traumas. Simple prosthetic foot was developed by Herbert and coworkers (71). Authors demonstrated that 3D printing is a simple and efficient technology of fabrication of prosthetic sockets that patients after amputation find comfortable. Another example was demonstrated by Zuniga who prepared low-cost 3D-printed hand for children with upper-limb reductions (72). Performed survey concluded that the prosthesis may have positive impact on the quality of life and can be applied for several activities in school and at home.

The 3D-printed structures may also help patients in revitalization and faster recovery after bone fractures as Cortex exoskeletal cast designed by Jake Evill. This orthopedic device in form of nylon mesh was light, durable and breathable, and what was described as the most important – water resistant (73).

Additive manufacturing is of particular attention in craniofacial reconstructive surgery as individual face features can be precisely replicated what strongly affects physical appearance and aesthetic issue. Congenital or traumatic deformities of ears are commonly replaced by silicone prosthesis or patients cartilage, however it is expensive and usually involves several visits in hospital. Moreover, it is difficult to obtain the shape that perfectly fit to the defect site without additional fillers or resection of healthy tissues. The 3D-printed ears were reported in 2014 (74). The main part was printed from polycaprolactone supported by PEG artificial layer and chondrocytes and adipocytes encapsulated in hydrogel. It was proven that the composite structure satisfied expectations for both the anatomy and geometry of the native ear. Fabrication of low cost soft tissue prostheses was described by He, Xue and Fu who applied Scanning Printing Polishing Casting (SPPC) to manufacture artificial ear (75). The tissue casting mold was prepared on the basis of 3D scan, chemically polished and casted with medical grade silicon. The fabrication of smooth prosthesis was estimated to cost ca. $30. Nasal prosthesis was obtained by Unkovskiy et al. who printed two silicon-based prostheses and applied post-process sealing and coloring (76). Both of obtained prostheses fitted well into the patient’s defect and set passively in position and both showed an acceptable adaptation to the adjacent skin.

The clinical benefits of 3D printing is also noticeable in the field of implantable medical devices. Nylon-based uretic stents and laparoscopic trocars were printed and successfully developed in a female cadaver and *in vivo* porcine model (77). While they fit the size of patients ureter, they exhibit some functional challenges resulted from limitations of currently available printers and materials.

The procedure of manufacturing of implants is usually based on digital images obtained using computed tomography (CT) or magnetic resonance imaging (MRI). The Computed Aided Design (CAD) is then conjugated with 3D printing and tailor-made structures are manufactured layer by layer. Such methodology can be particularly helpful for manufacturing of non-standardized implants for pediatric patients as described for tracheobronchomalacia treatment (78). Customized, collapse-resistance PCL-based bioresorbable tracheal splint was implanted in an infant.

Beside polymer-based implants, 3D-printed devices made of metals were also reported to be successfully implemented. First effort in manufacturing of personalized titanium plates implanted after microwave ablation used in bone tumor around the knee was described by Ma et al. (79) The authors described the process of implantation following tumor resection. The post-surgical evaluation of implant parameters as well as knee functionalities showed no fractures or loosening problems. Moreover, the mean maximum flexion of the knees was improved. The use of 3D-printed titanium plates was concluded as significant improvement in the surgical removal of bone tumor. Another customized implant was developed for the treatment of sternal tumor (80). Titanium implant was printed using direct metal laser sintering technology and applied for the reconstruction of chest wall defects. It was found to reduce morbidity and being beneficial for the patient in terms of optimal function and structural chest configuration after resection.

### Models for Surgical Planning and Training, Phantoms

Medical phantoms stay as highly demanded structures supporting the diagnosis and treatment of many diseases. While image-driven surgery has been widely used for decades, the need for rendering digital images has grown. The use of additive manufacturing of models enables more accurate diagnosis, better evaluation and assessment of the pathological changes as well as visualization of patient-specific organ anatomy. The preoperative planning significantly increases the amount of information beyond individual organ features what reduces the complications and patient loss (81). Thus, education and surgical planning is believed to be one of the most investigated field of 3D printing technology.

One of the example of using 3D-printed medical models is manufacturing of liver models. The growing demand for transplantations combined with the limited number of cadaveric livers increased the need of using the organ from healthy donors. The preoperative identification of the anatomy of vascular system and biliary tract can increase the safety of both donor and recipient. Transparent models with appropriate volume and color-coded vasculature was described in 2013 by Zein and coworkers (82). They prepared six models of livers of 6 patients and demonstrated identical anatomical and geometrical features between printed and native organs.

The 3D printing enables to plan the operation of resection of tumor as described in patient with colorectal liver metastasis (83). The CT scans were used to visualize the 3D anatomy of patient’s liver, and organ contour-defining parts, blood vessels as well as the tumor were printed. The model was then divided into 4 parts due to the printer limitations. Multilayered structure was assembled and filled with silicone. The production of resulted life-sized liver model with a transparent parenchyma, color-coded vascularization and tumor inside was estimated to cost less than $150. The patient underwent successful subsequent laparoscopic right hemihepatectomy. Another example of preoperative planning based on 3D-printed phantoms is modelling of renal malignancies (84). Stereolithography additive manufacturing was applied to manufacture 5 physical phantoms of clear translucent renal units with red translucent suspected malignancies. All patients underwent successful partial nephrectomy.

Silicon models of kidney, renal pelvis and ureter were developed in order to train pediatric laparoscopic pyeloplasty (85). Smaller workspace, finer sutures and more delicate tissues in comparison with adults make this operation challenging thus training is highly demanded. Printed models were tested with medical-imaging modalities such as magnetic resonance imaging, and 2D and 3D ultrasound. They were concluded to be useful in image-guided tumor resection or as platforms for surgical robotics. Moreover, the cost of production did not exceed $100 and several hours of labor.

Recently, additively manufactured models have been introduced into cardiology, especially concerning the teaching of congenital heart disease affected 1–2% of the world’s population and being the leading cause of mortality among infants in the US. These pediatric models have tremendous educational value, demonstrate complex anatomical concepts such as double-outlet right ventricle, malalignment-type ventricular septal defects and the spectrum of heterotaxy syndromes (86).

Aortic diseases treatment is easier due to the 3D-printed models that precisely reproduce aortic diameters and anatomy (87). Additive manufacturing of phantoms has been successfully introduced in patients with cardiac tumors or hypertrophic cardiomyopathy where the lesion size is the key parameter determining the selection of the surgical strategy, i.e. partial or total resection and heart transplant. This methodology has been implemented in the total resection of right ventricular. Implementation of 3D models is believed to provide rapid understanding of anatomical heart defects, including complex phenomena such as criss-cross atrioventricular connections (88).

The rising amount of patients suffering from degenerative diseases was also a problem addressed by additive manufacturing. Marks and coworkers showed 3D–printed brain in several stages of Alzheimer’s disease as an educational materials able to facilitate understanding of progressive degenerative changes in cerebral cortex and hippocampus (89). MRI images of brains of 5 patients in different clinical categories were captured and printed from 3D structures rendered after segmentation. The time of printing of each model varied from 15 to 20 h. The authors concluded that their models cannot be used for diagnosis but only for education purpose so far as some parameters need to be validated.

### Bioprinting and Organs-on-Chip

Aging-, disease- or accident-related organ failures are a serious medical problems in recent year. While transplantation serves as frequently used medical procedure, there is still the risk of rejection and lifelong immunosuppressant-base therapy. Thus, regenerative medicine has searched for a better solution. Recently, fabrication of multilayer object made from soft biological materials such as living cells is believed to be one of the most challenging and most promising technology. The future of patient-centered medicine is now explored in term of the development of biological constructs that are able to restore tissue architecture and functions (90).

So far, additive manufacturing has been mainly utilized for preparation of tissue construct such as cartilage, bone, blood vessels, liver, kidneys or heart tissue. Bioink containing alginate and nanocellulose was used to obtain structures representing cartilage tissue (91). A homogenous distribution of the cells was observed in printed material, however the shear forces and cross-linking affected cell viability. Rapid prototyping technology was also applied to utilize liver construct. A modular gelatin and hyaluronic acid-based hydrogel was used as a liver-specific bioink containing primary liver spheroids (92). Biofabricated tissue constructs exhibited high cell viability and ability to mimic *in vivo* liver functions.

To decrease the risk of rejection of 3D-printed construct autologous source of cell can be used. They may be either taken during biopsies or differentiation of autologous stem cells. The 3D-printed mesenchymal stem cell laden was described in the treatment of myocardial infarctions due to the ability of the cells to reduce the myocardial fate of postinfarction collagen deposition and scar tissue formation (93). However, therapeutic efficacy is limited by restricted transport of cell-secreting therapeutic cytokines at the implantation site. The developed cross-linked poly(ethylene glycol) dimethacrylate-based microchanneled hydrogel patch was found to effectively remove gel−tissue interface being a physical barrier for biomolecular transports thus facilitating the transport of cytokines.

Recently, numerous studies have been addressed to the high integration and dynamic changes in cell environment that affect tissue functions. Thus, microfluidics-based cell culture platform has been developed as an effective experimental tool in bioengineering of organs. A 3D-printed liver on-a-chip platform was developed using HepG2 and HUVEC cells and poly(ε-caprolactone) as a platform (94). One-step fabrication with heterotypic cell types and significantly enhanced functions of liver-on-chip was described. Microfluidics was also applied to print nervous system-on-chip as described by Johnson and co-workers as a model for the study of viral infection in the nervous system (95). Micro-extrusion 3D printing was utilized to manufacture microchannels for axonal alignment and compartmented chambers for cell isolation. The authors demonstrated *in vitro* functionality of personalized 3D-printed model and marked future perspective in the development of the treatment of neurological disorders.

Among numerous successful examples of printed bio-based objects, bioprinting still faces several problems. One of the most important issue is long-term viability of the cells. Another one is control of cell proliferation in order to provide sufficient amount of functional and supporting cells and tissue homeostasis. The choice of cell type is also important as they need to be able to reconstruct organ building blocks of various function, architecture and size. Moreover, the tissues used in 3D printing should be able to survive pressure and shear stress during printing, presence of harmful chemical compounds and nonphysiological pH. Beside aforementioned conditions, also scaffold material should meet given criteria such as biocompatibility, non-toxic by-products, appropriate mechanical and structural properties as this can affect cell migration, adhesion and proliferation (96).

### 4th Dimension of Printing

Considerable progress that has been made in the field of additive manufacturing led to the invention of smart 3D-printed materials able to change their shape and properties upon external stimuli over time. The so called 4D printing technology that considers manufacturing of shape memory materials, self-evolving structures and actuators for biorobotics has attracted considerable attention in recent years (97).

One of the earliest example of simple programmable mater is so called “mechanical protein” manufactured in MIT. These millimeter-size single-strain components were able to self-assembly into the letters MIT while the flat surfaces folded into a cube (98). More complex self-evolving materials were developed by Ravil who described 3D manufacturing of linear and ring stretching primitives as well as ring primitive composed of dynamic segments that reacts in a different way when exposed to water (99). The authors proved that self-deforming multi-purpose structures become one of the emerging technology.

4D printing has been also applied in the field of biomaterial manufacturing including biomedical devices, drug delivery and tissue engineering. The biomimetic ink containing cellulose fibril embedded in hydrogel matrix that exhibited localized swelling anisotrophy after immersion in water was described by Gladman (100). The authors described manufacturing of a series of shape-morphing systems that can be used in utilizing smart textiles or biomedical devices. The additive manufacturing of dynamically reconfigurable materials with tunable functionality was concluded to “open a new avenues for creating designer shape-shifting architectures for tissue engineering, biomedical devices, soft robotics and beyond”.

The 3D printing of stimuli-responsive materials has been also investigated in term of thermoresponsive hydrogels as they can be used to create drug delivery systems releasing the active compound upon thermal trigger at the first stage of inflammation. Poly(N-isopropylacrylamide) (PNIPAAm) hydrogel demonstrating the temperature-dependent swelling behavior was printed by means of projection micro-stereolitography. It was described as a promising material for further studies in the field of drug delivery vehicles (101). The same idea was presented by Ge et al. who described the properties of objects printed with tailorable shape memory methacrylate-based polymers. Obtained smart gels exhibited different thermomechanical behavior what was believed to poses the great potential in the development of scaffolds for cell growth and targeted delivery of small molecules such as water or drugs (102).

The 3D-printed core-shell capsules exhibiting programmable release was described by Gupta and co-workers. The capsules had aqueous cores and poly(lactic-co-glycolic) acid (PLGA) shells loaded with plasmonic gold nanorods which provided selective rapturing of the capsules upon laser irradiation. The capsules were described as a proof of concept of the combination of additive manufacturing and smart materials (103).

### Biorobotics

Bio-inspired hybrid devices being able to mimic various biological functions has recently been of considerable attention (104). The biorobots are constructed of artificial scaffold made from polymer elastomers or hydrogels that support soft biological matter such as proteins, living cells or tissues (105). They are more flexible than traditional robots and thus able to perform various types of movement such as walking or swimming and interact with surrounding environment. Within those robots rotary machines that are usually associated with transformation of chemical energy from the hydrolysis of ATP into work are the most inspiring (106). Cell-based actuators are usually cultured on thin flexible substrate. The contraction of the cells leads to film deflection and actuation as shown using mammalian cardiac and skeletal muscle cells (107).

Giving the advantages of 3D (bio)printing of tissues and organs the biorobots are highly demanded as they serve as small mechanical devices able to participate in tissue regeneration and drug delivery. They may also help to understand the locomotive mechanism of microorganisms as described by Williams and coworkers. They invented the flagellar swimmer with long tail and short head composed of polydimethylsiloxane (PDMS) filament cultured with cardiomiocytes (108). Moreover, the authors suggested that the method can be applied to other homotypic cell types, such as optogenetic muscle cells or heterotypic cells types, such as turtle cardiomiocytes and fibroblasts as well as neurons and muscle cells for sensing-based intelligent swimming. Another examples relies on seeding cardiomiocytes on PDMS membrane and formation of micro-spherical heart pump being able to control the flow within a microchannel due to the pulsatile motion of a diaphragm (109).

The biorobots are self-sufficient in energy and nutrients as the cells can generate the power to drive the motion and absorb sufficient components to maintain life processes. Although the viability of cells and control of motility still remains a challenge, some ideas to improve those factors have been introduced so far. They include enhancing the contraction force of the cells by applying anisotropic alignment, introducing electrical stimulation in order to control the rate of contractions or manufacturing of stimuli-responsive robots by incorporating light-sensitive cells (110). Manufacturing of biorobots by means of 3D bioprinting pave the way for the development of robust medical devices of tailor-made architecture and functionalities.

## Summary

The 3D printing of drug delivery systems and medical devices serves as an attractive tool to produce customized product. Since few years the concept of 3D-printed drug formulation quickly evolved and was directed to enhance therapy by patient-centric medicine. The first FDA approval of drug manufactured by 3D printing technology caused an exceptionally rapid development of studies on oral, oromucosal and topical dosage forms. This promising technology offers formulation flexibility that is difficult to achieve with the conventional technological processes. Additional manufacturing allows to prepare different kind of dosage forms with high precision of API-excipients ratio, in totally new manner with comparison to traditional pharmaceutical manufacturing. The added value of the 3D printing is also opportunity to create multifunctional drug delivery systems, multidrug devices and drug formulations for personalized therapy with accelerated release characteristic. Therefore, future research should prioritize the development of pediatric and geriatric dosage forms in personalized dosing and dimension-specific drug formulations to ensure desired therapeutic effect. Increasing amount of drug development studies proves undeniable benefits of this technology but the full success will be obtained after leading elaborated new dosage forms on an industrial scale.

The introduction of additive manufacturing into a clinic reduces the time and costs of medical treatment and improves the success rate of surgeries. It may also lead to the development of new surgical procedures, especially concerning these risky and rarely performed. Moreover, 3D printing of highly mimetic models of organs for surgical training can ease and shorten operation time and decrease the intra-operative complications.

The use of living cells enables formation of biomaterials for reproducing vascularized tissues that can be used for implantation, drug screening as well as disease modelling and cancer research. The development of biorobots opens a new scope of the development of sensors relying on physiological changes experienced by cells or even artificial immune system.

Giving all the advantages, additive manufacturing needs to face several challenges in terms of design parameters control, device performance, biocompatibility of printed material and sterilization. Furthermore the fragile nature of printed objects, especially cell-based combined with complex nature of manufactured structures requires well-planned procedure. However, all benefits for patients and overall healthcare system provided by the implementation of 3D printing makes the amount of required research leading to establishing the process of customized products manufacturing reasonable.

## ABBREVIATIONS

3DP: Three-dimensional printing
ABS: Acrylonitrile butadiene styrene
AM: Additive manufacturing
API: Active pharmaceutical ingredient
CAD: Computed aided design
CT: Computed tomography
dcDU: Dual-compartmental dosage unit
DLP: Digital light projector
DOD: Drop on drop
DOS: Drop on solid
DPPO: Diphenyl(2,4,6-trimethylbenzoyl)phosphine oxide
EC: Ethyl cellulose
FDA: Food and Drug Administration
FDM: Fused deposition modeling
HIPS: High impact polystyrene
HME: Hot melt extrusion
HPC: Hydroxypropyl cellulose
HPMC: Hydroxypropyl methylcellulose
HPMCAS: Hydroxypropyl methylcellulose acetate succinate
MRI: Magnetic resonance imaging
ODF: Orodispersible films
PCL: Polycaprolactone
PDMS: Polydimethylsiloxane
PEG: Poly(ethylene glycol)
PEGDA: Poly(ethylene glycol) diacrylate
PET-G: Polyethylene terephthalate glycol-modified
PLA: Polylactic acid
PVA: Poly(vinyl alcohol)
PVP: Polyvinylpyrrolidone
RP: Rapid prototyping
SLA: Stereolithography
SLM: Selective laser melting
SLS: Selective laser sintering
SPPC: Scanning printing polishing casting
T_g_: Glass transition temperature
